# Mortality and clinical outcomes in preterm infants with patent ductus arteriosus according to pharmacological treatment or expectant management: a retrospective cohort study

**DOI:** 10.3389/fped.2026.1952588

**Published:** 2026-10-08

**Authors:** Javier Torres-Muñoz, Diana María Pérez Manrique, Lesly Georjany Escobar Gordillo, Angie Marcela Guapacha Alomia, Julián Torres-Figueroa

**Affiliations:** 1 INSIDE Group Research, School of Medicine, Faculty of Health, Universidad del Valle, Cali, Colombia; 2 Department of Pediatrics, School of Medicine, Faculty of Health, Universidad del Valle, Cali, Colombia; 3 Versailles Clinic Cali, Cali, Colombia; 4 Faculty of Health Sciences, Universidad ICESI, Cali, Colombia

**Keywords:** low birth weight, mortality, patent ductus arteriosus, preterm newborn, risk factors, survival analysis

## Abstract

**Introduction:**

Patent ductus arteriosus (PDA) is frequent in preterm newborns and its management remains controversial. The objective was to evaluate the association between pharmacological treatment of PDA and mortality in preterm infants.

**Methods:**

Retrospective cohort study. A sample size of 154 patients was estimated. Comparisons were performed using the Chi-square test for qualitative variables, and the Mann–Whitney test for continuous variables. Survival analysis was performed using the Kaplan–Meier curve and log-rank test. Cox regression models were used to estimate Hazard ratios (HR) with 95% confidence intervals (95% CI).

**Results:**

A total of 156 patients were included. In the survival analysis, Kaplan–Meier curves showed a lower probability of survival in the treated group.

In the Cox proportional hazards model, pharmacological treatment was not independently associated with mortality (HR: 2.25; 95% CI: 0.86–5.88; *p* = 0.09). Birth weight was independently associated with a lower mortality hazard rate (HR: 0.997; 95% CI: 0.995–0.999; *p* = 0.020). No significant associations were observed with sepsis.

**Conclusion:**

Expectant management was not associated with an increase in morbidity or mortality. The lower survival observed with pharmacological treatment in the unadjusted analyses was not maintained after adjustment.

## Introduction

Patent ductus arteriosus (PDA) is a frequent condition in preterm newborns and is associated with significant morbidity and mortality rates that may reach 30% when it is hemodynamically significant (1, 2). In preterm infants younger than 28 weeks of gestational age and in those with a birth weight lower than 1,500 grams, the probability of spontaneous closure is lower, with estimates suggesting that more than 50% of these cases do not achieve spontaneous closure (3).

Despite advances in neonatal care, the optimal management of PDA continues to be controversial (2–4). Traditionally, the treatment of hemodynamically significant PDA in preterm infants has included the use of prostaglandin inhibitors, acetaminophen, and, in refractory cases, surgical closure (5). These interventions aim to prevent complications associated with PDA, such as pulmonary overload secondary to left-to-right shunting, intraventricular hemorrhage (IVH), necrotizing enterocolitis (NEC), bronchopulmonary dysplasia (BPD), and mortality (2).

However, in recent years, evidence has emerged questioning the direct causal relationship between persistent PDA and these complications, as well as the benefits of pharmacological treatment. In addition, potential adverse effects associated with these interventions have been described. In this context, expectant management of hemodynamically significant PDA has gained relevance, even in very low and extremely low birth weight newborns, leading some centers to adopt more conservative strategies (2, 6).

The non-interventionist approach has been evaluated in different studies, showing that it is not associated with an increase in mortality or in the main neonatal morbidities, provided that it is accompanied by adequate management, particularly of fluid balance (7, 8). Likewise, recent meta-analyses have reported a possible association between pharmacological treatment and higher mortality compared with expectant management (9). Similarly, the very early use of ibuprofen has been linked to a moderate increase in mortality in preterm infants younger than 28 weeks of gestation (10).

In this context, current evidence raises the need to reevaluate therapeutic strategies in the management of hemodynamically significant PDA, prioritizing interventions that minimize harm and optimize clinical outcomes. This is especially relevant in low- and middle-income settings, where the implementation of evidence-based protocols may have a significant impact on the survival and evolution of these patients.

## Methodology

A retrospective cohort study was conducted in preterm newborns between 26 weeks and 0 days and 32 weeks and 6 days of gestational age, according to the best obstetric estimate, or with a birth weight lower than 1,500 grams. The included patients were born and admitted to the neonatal intensive care units of two medical institutions between April 1, 2023 and December 31, 2024. These institutions attend approximately 60% of births in the city of Cali.

Newborns with a diagnosis by pediatric cardiologist echocardiography of hemodynamically significant PDA after 72 h of life were included. A PDA was considered hemodynamically significant when it presented with left to right flow, ductal diameter ≥ 1.5 mm, and LA/Ao ratio ≥ 1.5, associated with at least one sign of pulmonary over circulation or systemic hypoperfusion, such as reversed diastolic flow in the descending aorta or increased respiratory support (11). In both participating institutions, a standardized echocardiographic surveillance protocol was used for these patients. This protocol involves performing follow-up echocardiograms on a weekly basis, with an additional evaluation at hospital discharge when the patient's clinical condition warrants it.

The proportion of infants who received ibuprofen versus paracetamol was not systematically recorded as a study variable and, therefore, could not be reliably reported. No specific drug was predefined as a first-line treatment. The choice between ibuprofen and paracetamol was based on drug availability and the clinical judgment of the attending neonatologist, in accordance with institutional protocol and each infant's clinical characteristics. The intravenous ibuprofen regimen consisted of 10 mg/kg on day 1, followed by 5 mg/kg on days 2 and 3. The intravenous paracetamol regimen consisted of 15 mg/kg per dose every 6 h for 3 days.

Initially, 233 preterm infants were identified (Figure 1). Of these, 77 were excluded: 16 for having a gestational age between 23 weeks and 25.6 weeks, one for presenting a major congenital malformation, and 60 for not being identified with PDA. Newborns with gestational age between 23 and 25.6 weeks were excluded due to the limited number of available patients and the high early mortality observed in this group in our setting during the study period, which precluded comparable analyses and robust statistical estimates. Furthermore, this subgroup presents substantially different clinical characteristics and therapeutic strategies, with a higher incidence of hemodynamically significant PDA and a greater likelihood of receiving pharmacological or interventional treatment. Its inclusion would have increased the heterogeneity of the study population and could have introduced significant confounding bias. We have incorporated this clarification into the methods section and acknowledged as a limitation that the results are not directly extrapolable to extremely preterm newborns (<26 weeks).

**Figure 1:**
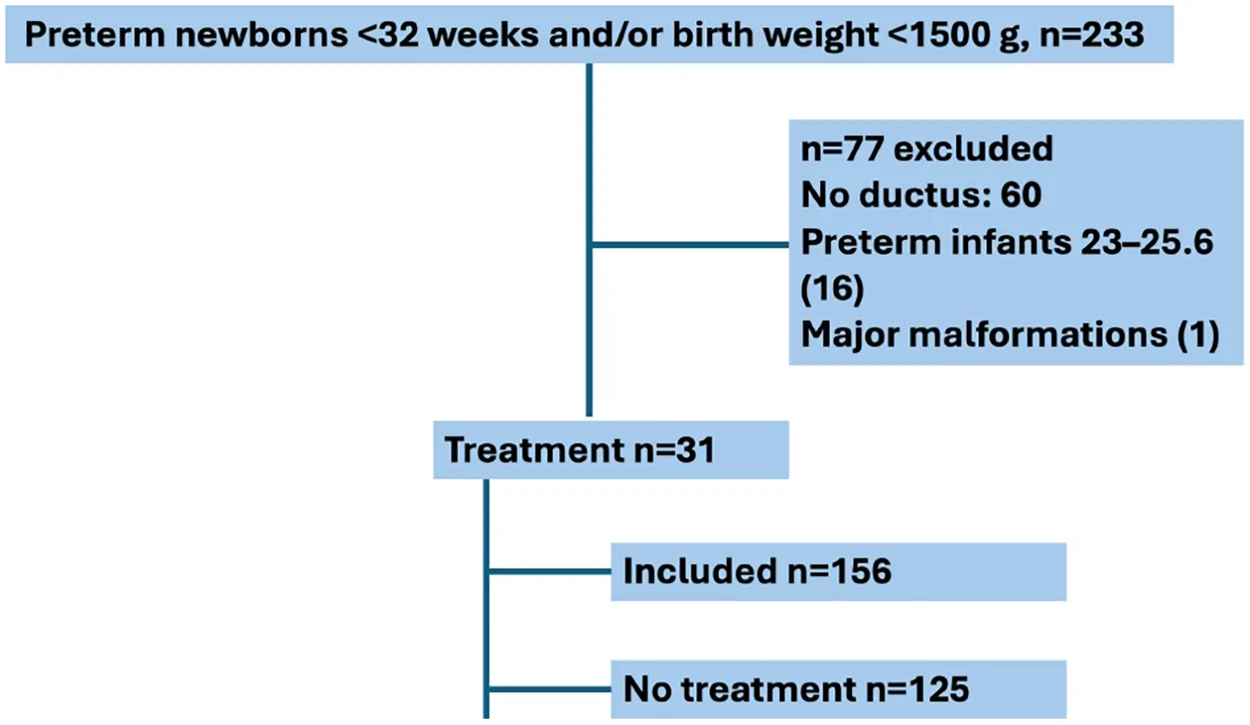
Total number of patients evaluated during the study; 2023–2024, infants born at <32 weeks of gestation and/or weighing <1,500 grams at birth, admitted to neonatal intensive care units.

The sample size calculation was performed taking as reference the study by Borras et al. (11) in Brazil in preterm infants between 25 and 32 weeks of gestational age, in which the maximum estimated sample size refers to the one calculated with the risk factor associating pharmacological treatment of PDA versus conservative treatment. The proportion of those exposed to medical treatment was 40% and unexposed 60%, with an alpha error of 0.1 and a beta error of 0.20. A sample size of 77 exposed and 77 unexposed to medical treatment was determined, for a total sample of 154 patients using the Epidat version 4.1 program.

The sample size calculation was performed based on the study by Borras et al. (12), using an expected risk of 40% in the exposed group and 60% in the unexposed group, with a risk ratio of 0.667, a 1:1 ratio of unexposed to exposed participants, with a 90% confidence level and 80% statistical power. The resulting minimum sample size was 154 participants (77 exposed and 77 unexposed), calculated using Epidat version 4.1. This allocation represented the ratio used for the sample size calculation and did not correspond to the actual distribution of treatment in the study. All consecutive eligible infants admitted during the study period were assessed for inclusion, regardless of PDA management. A total of 156 eligible infants were ultimately included, comprising 127 infants managed expectantly and 29 who received pharmacological treatment.

Demographic and clinical variables were collected, including timing of echocardiography performance, identification of hemodynamically significant PDA according to the pediatric cardiologist’s criteria, administration of pharmacological treatment which included ibuprofen/acetaminophen, administered according to institutional protocols (at the time of diagnosis), time during which pharmacological treatment was received, time at which the PDA closed, PDA status at discharge, surfactant use, bronchopulmonary dysplasia (defined as oxygen requirement at 36 weeks of corrected gestational age) (13), severe intraventricular hemorrhage (grades III and IHPV) (14)), necrotizing enterocolitis (modified Bell stage II or higher) (14), retinopathy of prematurity greater than stage 2 (15), duration of oxygen requirement, and survival to hospital discharge.

Data were collected retrospectively from the databases of the neonatal units during the hospitalization period.

## Outcomes

The primary outcome was mortality before hospital discharge. Secondary outcomes included bronchopulmonary dysplasia (BPD), necrotizing enterocolitis (NEC), retinopathy of prematurity (ROP), intraventricular hemorrhage (IVH), sepsis, duration of supplemental oxygen, length of hospital stay, PDA closure during hospitalization, time to PDA closure. PDA closure by echocardiography (days), Birth weight (g), Gestational age (weeks).

Infants were classified according to PDA management into two groups: (1) **expectant management**, defined as no pharmacological treatment for PDA, and (2) **pharmacological treatment**, defined as treatment with ibuprofen and/or paracetamol according to institutional protocols. No infant underwent surgical or transcatheter PDA closure during the study period.

## Statistical analysis

Continuous variables were assessed for normality using the Shapiro–Wilk test. Normally distributed variables were summarized as means and standard deviations, whereas non-normally distributed variables were presented as medians and interquartile ranges. Categorical variables were summarized as absolute frequencies and percentages.

Continuous variables were compared between the expectant management and pharmacological treatment groups using Student's *t*-test or the Mann–Whitney *U* test, as appropriate. Categorical variables were compared using the chi-square test or Fisher's exact test.

Multivariable logistic regression models were used to evaluate the association between PDA management and binary clinical outcomes. Adjusted odds ratios (ORs) with 95% confidence intervals (CIs) were reported. Potential confounders considered for adjustment included gestational age, birth weight, sex, Apgar score, and resuscitation at birth. Covariates were selected based on clinical relevance and their potential role as prognostic factors or confounders rather than solely on statistical significance in univariable analyses.

Time-to-event analyses were performed using Kaplan–Meier methods. For mortality, survival time was measured from the defined starting point to death or censoring. Survival curves according to PDA management were compared using the log-rank test, and the number of patients at risk was reported at prespecified time points.

Cox proportional hazards regression was used to evaluate the association between PDA management and time to death. Univariable Cox regression models were initially fitted separately for each candidate covariate. Covariates included in the multivariable models were selected *a priori* for each outcome based on clinical relevance, biological plausibility, and their potential role as prognostic factors or confounders. Selection was specific to each outcome and statistical model and was not based solely on statistical significance in univariable analyses. For the mortality analysis, the multivariable Cox model included PDA treatment, gestational age, birth weight, and sepsis. No automated stepwise variable-selection procedure was used.

Hazard ratios (HRs) with 95% confidence intervals (CIs) were reported. The proportional hazards assumption was assessed using Schoenfeld residual-based tests after fitting the multivariable Cox model. A two-sided *p* value <0.05 was considered statistically significant.

All analyses were performed using Stata version 14 (StataCorp, College Station, TX, USA).

## Results

A total of 156 patients were included in the analysis (Figure 1). The characteristics of the studied population are presented in Table 1. No significant differences were observed between the two groups (received treatment or not) regarding the use of prenatal steroids and maternal morbidity. The median age at initiation of pharmacological treatment was 8 days (IQR: 5-12 days). Infants who received pharmacological treatment had a significantly lower gestational age (*p*=<0.001), with a median of 27 weeks (IQR: 26.2–28.1), compared with 30 weeks (IQR: 28.1–31.5) in the untreated group. Likewise, they had significantly lower birth weight (*p* < 0.001), with the median of 885 grams (IQR: 775-1,110) versus 1,284 grams (IQR: 1100-1493) in the untreated group (Table 2).

**Table 1:** Initial exploration of sociodemographic, maternal, and neonatal variables in preterm infants younger than 32 weeks and/or with birth weight <1,500 g, according to treatment for patent ductus arteriosus (PDA) in two institutions in Cali, 2023–2024.

Variable	No. /total no. (%) of patients		*P* value
	**No treatment group (*n*** **=** **127)**	**Active treatment group (*n*** **=** **29)**	
Variable			
Female gender	72/127 (56.7)	14/29 (48.3)	0.41
Prenatal care ≤3	16/125 (12.8)	6/29 (20.6)	0.27
Maternal age Age ≤19 years	16/124 (12.9)	4/28 (14.2)	0.84
Primigravida	57/122 (46.7)	8/25 (32)	0.17
Gestational age ≤ 28 weeks	24/127 (18.9)	18/29 (13.3)	<0.01
Birth weight <1,000 gr	17/127 (13.4)	19/29 (65.6)	<0.01
No Antenatal steroids	23/127 (18.1)	7/29 (24.1)	0.45
Cesarean section	112/127 (88.2)	27/29 (93.1)	0.44
Neonatal resuscitation	88/127 (69.3)	26/29 (89.6)	0.02
Use of caffeine	68/127 (53.6)	4/29 (13.8)	<0.01
PDA status open at discharge	24/109 (22.0)	8/28 (28.6)	0.46
Bronchopulmonary Dysplasia	32/122 (26.2)	16/27 (59.3)	<0.01
Necrotizing enterocolitis	12/127 (9.4)	7/29 (24.1)	0.02
Intraventricular hemorrhage	24/127 (18.9)	7/29 (24.14)	0.52
Retinopathy	16/118 (13.5)	6/29 (20.7)	0.33
Sepsis	50/127 (39.4)	19/29 (65.5)	0.01
Mortality	10/127 (7.8)	21/29 (72.4)	<0.01
Length of stay >15 days	112/127 (82.2)	24/29 (82.8)	0.43

Values are expressed as *n*/*N* (%). that denominators vary because of missing data. A *p* value < 0.05 was considered statistically significant.

**Table 2:** Analysis of continuous variables in preterm infants <32 weeks and/or <1,500 g according to treatment for patent ductus arteriosus (PDA). Cali, 2023–2024.

Variable	No treatment		Treatment		*p* value
	Median	IQR (25–75)	Median	IQR (25–75)	
Duration of supplemental O₂ (days)	17	6–43	63	22–97	<0.01
Days to PDA diagnosis	7	4–7	4	4–7	0.29
PDA closure by echocardiography (days)	17.5	7–32	15	9.5–22.5	0.64
Length of hospital stay (days)	33	20–50	66	23–102	<0.01
Birth weight (g)	1,284	1,100–1,493	885	775–1,110	<0.001
Gestational age (weeks)	30	28.1–31.5	27	26.2–28.1	<0.001

Statistically significant differences were found in gestational age and birth weight between the groups according to treatment administration (Mann–Whitney test, z = 5.40; *p* < 0.001), with higher values observed in the group that did not receive treatment.

In the unadjusted Kaplan–Meier analysis (Figure 2), survival was significantly lower in the pharmacological treatment group (log-rank *χ*^2^ = 10.37; *p* = 0.0013).

**Figure 2:**
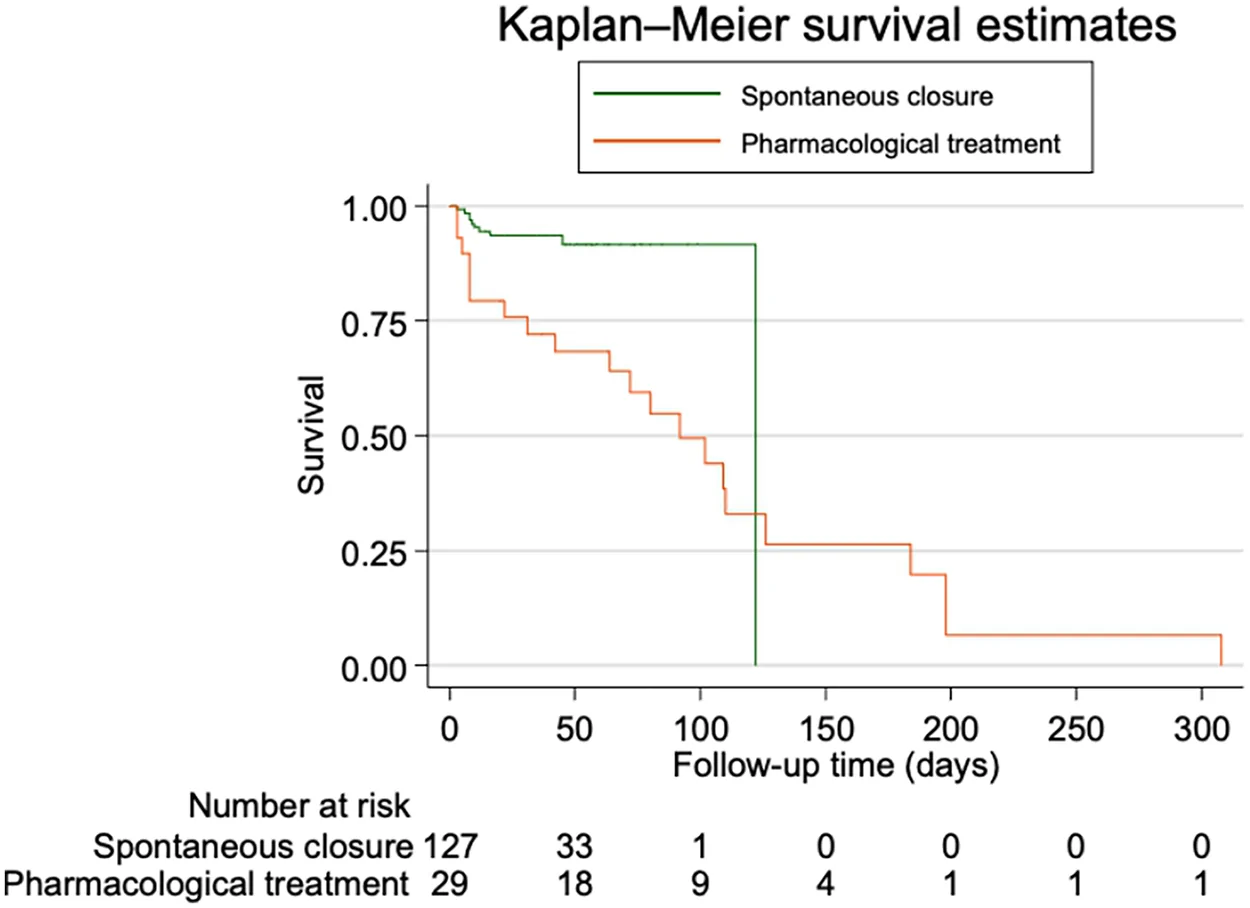
Unadjusted Kaplan–Meier survival curves according to management of patent ductus arteriosus. Infants who received pharmacological treatment had significantly lower survival than those managed expectantly (log-rank test, *χ*² = 10.37; *p* = 0.0013). There were 21 deaths in the pharmacological treatment group and 10 deaths in the spontaneous closure group. The number-at-risk table is shown below the curves.

In the univariable Cox regression analysis, pharmacological treatment was associated with a higher hazard of mortality (HR 4.01, 95% CI 1.67–9.63; *p* = 0.002). Birth weight was also associated with mortality (HR 0.997 per gram, 95% CI 0.996–0.999; *p* = 0.001).

After multivariable adjustment for gestational age, birth weight, and sepsis, the association between pharmacological treatment and mortality was attenuated and was no longer statistically significant (HR 2.25, 95% CI 0.86–5.88; *p* = 0.097). Birth weight remained independently associated with mortality (HR 0.998 per gram, 95% CI 0.996–0.999; *p* = 0.040), whereas gestational age and sepsis were not significantly associated with mortality (Table 3).

**Table 3:** Survival analysis: adjusted cox proportional hazards model.

Variable	AdjustedHR	CI 95%	*p* value
Pharmacological treatment	2.25	0.86–5.88	0.09
Birth weight (per 100 g)	0.997	0.995–0.999	0.02
Age gestational	0.85	0.38–1.89	0.69
Sepsis	0.99	0.36–2.73	0.98

HR, hazard ratio; 95% CI, 95% confidence interval. Birth weight was modeled in increments of 100 g.

The proportional hazards assumption was satisfied for all covariates, with no evidence of violation for treatment (*p* = 0.930), gestational age (*p* = 0.714), birth weight (*p* = 0.869), or sepsis (*p* = 0.419). The global Schoenfeld residual test was also consistent with the proportional hazards assumption (*χ*^2^ = 1.02, df = 4, *p* = 0.908). (Tabla 3) Regarding the timing of death, the median age at death was 31 days (IQR, 8–109 days). There were 21 deaths among infants who received pharmacological treatment and 10 among those managed expectantly. The causes of death were not systematically collected as a study variable and therefore were not available for analysis.

The multivariable Cox model should be interpreted cautiously because only 31 deaths occurred among 156 infants and five covariates were included in the model. This limited number of events may have resulted in some degree of overfitting and imprecision.

## Discussion

In this study, we found that expectant management of patent ductus arteriosus (PDA) in preterm infants younger than 32 weeks was not associated with higher mortality or with increased rates of morbidities.

However, these findings should be interpreted in the context of the substantial baseline differences between treatment groups. Infants who received pharmacological treatment had lower gestational age and birth weight, suggesting substantial confounding by indication. Although lower survival was observed among pharmacologically treated infants in the unadjusted analysis, this association was no longer statistically significant after multivariable adjustment. Therefore, the observed difference in unadjusted survival should not be interpreted as evidence of a detrimental effect of pharmacological treatment, but rather may reflect differences in baseline clinical severity between groups.

These findings are consistent with recent evidence, which indicates that pharmacological treatment of PDA continues to be a controversial topic in neonatology. In this regard, some authors have proposed using the term “attempt to accelerate closure” instead of “treatment,” given the limited evidence regarding its impact on clinically relevant outcomes (16–19).

In our study, expectant management was not associated with an increase in morbidity or mortality. The lower survival observed in the treated group in the unadjusted analyses lost significance after multivariate adjustment, suggesting the influence of indication bias rather than a causal effect of treatment.

In agreement with these results, studies in extremely low birth weight preterm infants have described higher mortality in the treated group, especially in those younger than 29 weeks (9). Likewise, a Bayesian meta-analysis (20) reported a possible increase in the risk of BPD or the composite outcome of BPD or death in the treated group, particularly in the most immature patients. However, the evidence is not uniform, since other studies have not found significant differences between management strategies in outcomes such as mortality (21).

The TREOCAPA study (22), which evaluated the use of paracetamol in preterm infants between 23 and 28 weeks, did not demonstrate a benefit in survival free of severe morbidity, despite favoring ductal closure. These results suggest that anatomical closure of PDA does not necessarily translate into clinical benefits.

On the other hand, studies evaluating the natural evolution of PDA have shown that conservative management does not increase mortality or morbidity, even in the most extremely preterm infants (8, 23). Although spontaneous closure may be delayed in the most immature patients, this is not associated with worse clinical outcomes (24).

Taken together, our results and the available evidence suggest that expectant management of PDA is a safe strategy in many cases.

## Conclusion

Expectant management of patent ductus arteriosus was not associated with an increase in morbidity or mortality. Pharmacological treatment was associated with lower survival in the unadjusted analyses, but not independently after adjustment, suggesting the influence of baseline clinical severity and indication bias.

Among the strengths of the study are the use of survival analysis and multivariable models. However, its retrospective design limits causal inference and does not exclude the presence of residual confounding, particularly due to unmeasured variables related to clinical severity. In addition, possible selection bias and the sample size may affect the precision of the estimates. Prospective studies are required to confirm these findings.

## Data Availability

The original contributions presented in the study are included in the article/Supplementary Material, further inquiries can be directed to the corresponding author.
